# Assessing the implications of habitat transformations on human-large carnivore interactions outside protected areas

**DOI:** 10.1038/s41598-025-13808-4

**Published:** 2025-08-06

**Authors:** Vivek Ranjan, Ruchi Badola, Syed Ainul Hussain, Parag Madhukar Dhakate

**Affiliations:** 1 Wildlife Institute of India, Chandrabani, Dehradun, 248002 Uttarakhand India; 2 Uttarakhand Forest Department, Rajpur Road, Dehradun, 248001 Uttarakhand India

**Keywords:** Hotspot, Land use, Human-wildlife conflict, Terai landscape, Wildlife corridor, Conservation biology, Forest ecology, Forestry, Tropical ecology, Ecology, Ecology, Biodiversity, Ecosystem ecology

## Abstract

Humans and wildlife have coexisted spatially and temporally for many years. However, this is disturbed when human-induced changes constrain limited and shared resources, leading to increased competition for resources and negative human-wildlife interactions. This study aims to examine the implications of habitat changes on human–large carnivore interaction (HLCI) and identify priority areas for negative interaction. The study was conducted in the Terai Arc landscape in two study blocks constituting three wildlife corridors in the Corbett and Indo-Nepal transboundary landscapes. This study assessed the decadal changes in habitats and identified priority sites of negative interaction based on reported wildlife attacks due to *Panthera tigris* and *Panthera pardus*. The study mapped five spatial risk zones, from very low to very high, and determined the effects of habitat changes on HLCI. Changes in land-use patterns, vegetation health, and anthropogenic activity have significant effects on hotspots of negative HLCI. Our results will support decision-makers and park managers in streamlining, managing, and prioritizing local and regional mitigation efforts at the identified sites. This approach will help improve the co-existence scenario in the landscape.

**Supplementary Information:**

The online version contains supplementary material available at 10.1038/s41598-025-13808-4.

## Introduction

Humans and wildlife have been coexisting and sustaining their life cycles close to each other since time immemorial. The nature of human-wildlife co-existence depends upon their interaction, which may range from positive to neutral to negative^1^. It is a negative interaction where anthropogenic losses or losses of wildlife species occur, generally referred to as conflict or human-wildlife conflict (HWC)^2–4^. Accommodating human needs and wildlife in a shared natural space has been considered one of the most significant and fundamental conservation challenges of the 21 st century^5–7^. The communities living close to forest habitats have a high probability of human-wildlife interaction (HWI)^6^. Rapidly changing demographic, socioeconomic, and environmental factors significantly influence the occurrence and patterns of HWI^8^.

The terai landscape has also experienced rapid population increase and land-use changes over time, like encroachment of habitats for agricultural uses, croplands transforming into built-up and industrial areas, and an increase in linear infrastructures^9,10^. As per the Census of India 2011, the population growth has shown an exponential curve in the Terai region of Uttar Pradesh (U.P.) and Uttarakhand over the last 100 years, the decadal variation shows a population increase of more than 400%. The Nainital district of Uttarakhand, which also encompasses part of our study area, shows a population growth from 1,82,016 in 1911 to 9,54,605 in 2011; another district from the Terai area of Uttarakhand, i.e., Udham Singh Nagar also borders with the state of U.P and one of the most industrialised districts shows a population increase of more than 11 times of the population in 1911.

Rapidly increasing human populations and their needs have led to large-scale land-use changes. Landscape-level changes and the transformation of habitats are significant concerns for large and wide-ranging mammals such as tigers, leopards, and elephants. One vital tool for their long-term conservation is establishing protected areas (PAs) as secured natural systems to conserve flora and fauna^11^. The global network of PAs accounts for 17% of the total terrestrial surface area^12^. However, these PAs are often fragmented, isolated, and disconnected from adjoining non-protected forest areas in a human-dominated mosaic landscape^11,13^. Assuring the prosperity and peaceful co-existence of communities living with wildlife and on the fringe of these PAs is a significant challenge for sustainable development^14,15^. The culture and traditional way of life in Uttarakhand supports co-existence and tolerance towards wildlife. However, in current scenario of increasing anthropogenic pressure on forests and loss of traditional ways of subsistence has led to strenuous conditions for human-wildlife co-existence, sometimes turning to conflict due to negative HWI.

Protecting wide-ranging large carnivores is difficult because they frequently prey on valuable livestock on community lands and areas close to protected areas^14,16,17^. Large carnivores are territorial species and occur at low population densities, requiring vast extant habitats, thus becoming more vulnerable to habitat loss^18^. Studies have highlighted the importance of multi-use landscapes for conserving large carnivores, especially in regions where carnivore ranges overlap with high human-density areas^11,19^. One of the critical tasks in carnivore conservation is identifying priority human-carnivore conflict sites and their underlying reasons for effective mitigation strategies^20^.

This study investigates the implications of habitat transformations on the Human–Large Carnivore Interaction (HLCI), as we hypothesise that human-induced habitat changes are one of the critical drivers and factors for negative HWI. Thus, this study focuses on the changes in the habitat attributes of the wildlife corridors and adjoining areas and their influence on negative HLCI. We assume that rapid changes in habitat features and quality induces unfavourable conditions for large mammals, increasing the chances of negative interaction with humans. This study identifies priority hotspot sites for leopard and tiger-related negative interactions as well as overall for large carnivores, i.e., tigers (*Panthera tigris*) and leopards (*Panthera pardus*) combined, based on the occurrence of negative HWI events for specific species. We examined the effects of land-use/land cover (LULC) changes and other habitat modifications over time on negative HLCI hotspots. Habitat transformations affect the ecology of species in various ways^21–23^. Even sympatric species of similar size and behavior respond differently to human stressors^24^. Thus, we also explored the implications of sympatric large carnivorous species on the spatial hotspots of a particular species, along with habitat implications. Understanding the relationship between habitat modifications and HWI is essential to strategize the effective and efficient long-term conservation and co-existence of humans and large mammals.

## Results

The data listed five species (Tiger, Leopard, Elephant, Wild Boar, Sloth Bear) and two taxa (deer and snakes) of wildlife species involved in the HWC as per the incidents reported to the Uttarakhand Forest Department. The incidents reported for large carnivores, i.e., tigers and leopards, numbered 1466 from 2006 to 2020, where Block 1 had 1060 incidents from 2009 to 2020, and Block 2 had 406 incidents reported from 2006 to 2020. Two types of negative interactions are observed with large carnivores: livestock depredation and human casualties (injury and killing). In Block 1, 48% of the cases involved tigers, 7% involved leopards, and 45% did not mention species due to a lack of confirmed identification. In Block 2, 14% of the cases were related to tigers, 36% were leopards, and 50% were without confirmed species. In study block 1, 14 cases of human casualties were reported, of which 10 were due to tigers, and four were due to leopards. In study block 2, there were 25 cases of human casualties, 19 cases due to tigers and six to leopards. Livestock depredation incidents involve five livestock species, i.e., cows, buffaloes, ox, goats, and horses. Leopards also kill domestic or stray dogs, but such cases were not compensated and thus were out of the purview of this study. The lack of awareness and inconsistency in reporting the species involved in incidents pose serious issues in the comparative analysis of data between tiger and leopard conflict patterns.

### Spatial risk mapping

**Fig. 1:**
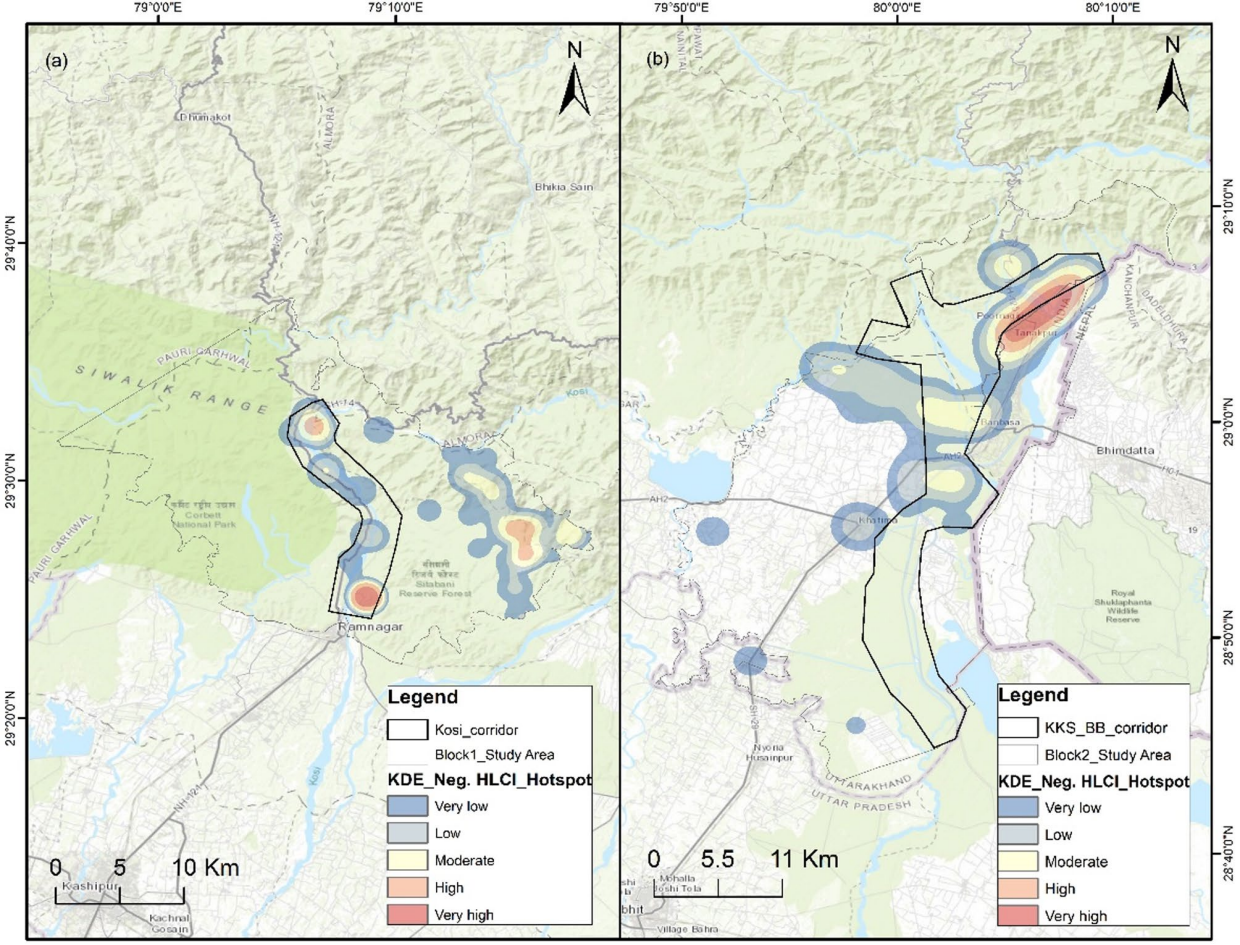
Spatial risk hotspot map of negative HLCIs with five risk zone categories in the study area. (**a**) Study block 1 constitutes the Kosi corridor connecting the CTR to the Ramanagar FD and Pawalgarh Conservation Reserve. (**b**) Study block 2 comprises the KKS and BB wildlife corridor networks. (Neg. HLCI: Negative Human Large Carnivore Interaction, CTR: Corbett Tiger Reserve, FD: Forest Division, KKS: Kilpura-Khatima-Surai, and BB: Boom-Brahmadev). The map was generated in ArcGIS Desktop 10.7.0 (Esri, Inc.) software and used India Topographic map as basemap; Service Layer Credits: Sources: Esri, HERE, Garmin, Intermap, increment P Corp., GEBCO, USGS, FAO, NPS, NRCAN, GeoBase, IGN, Kadaster NL, Ordnance Survey, Esri Japan, METI, Esri China (Hong Kong), © OpenStreetMap contributors, and the GIS User Community.

**Fig. 2:**
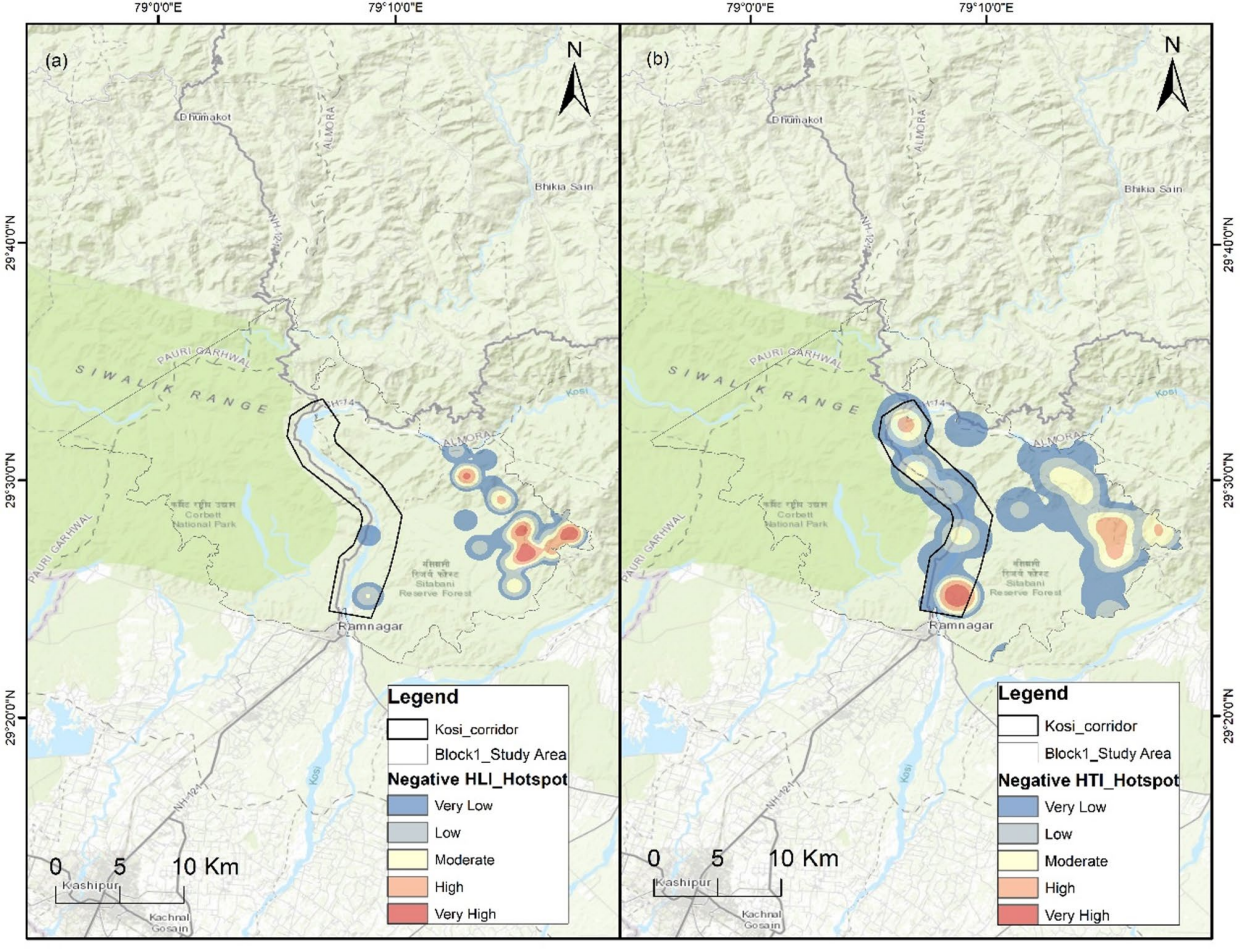
Spatial risk hotspot map of Study Block 1, which is composed of the Kosi Corridor. (**a**) Negative Human-Leopard Interaction (HLI) Hotspots (**b**) Negative Human-Tiger Interaction (HTI) Hotspots. The map was generated in ArcGIS Desktop 10.7.0 (Esri, Inc.) software and used India Topographic map as basemap; Service Layer Credits: Sources: Esri, HERE, Garmin, Intermap, increment P Corp., GEBCO, USGS, FAO, NPS, NRCAN, GeoBase, IGN, Kadaster NL, Ordnance Survey, Esri Japan, METI, Esri China (Hong Kong), © OpenStreetMap contributors, and the GIS User Community.

**Fig. 3:**
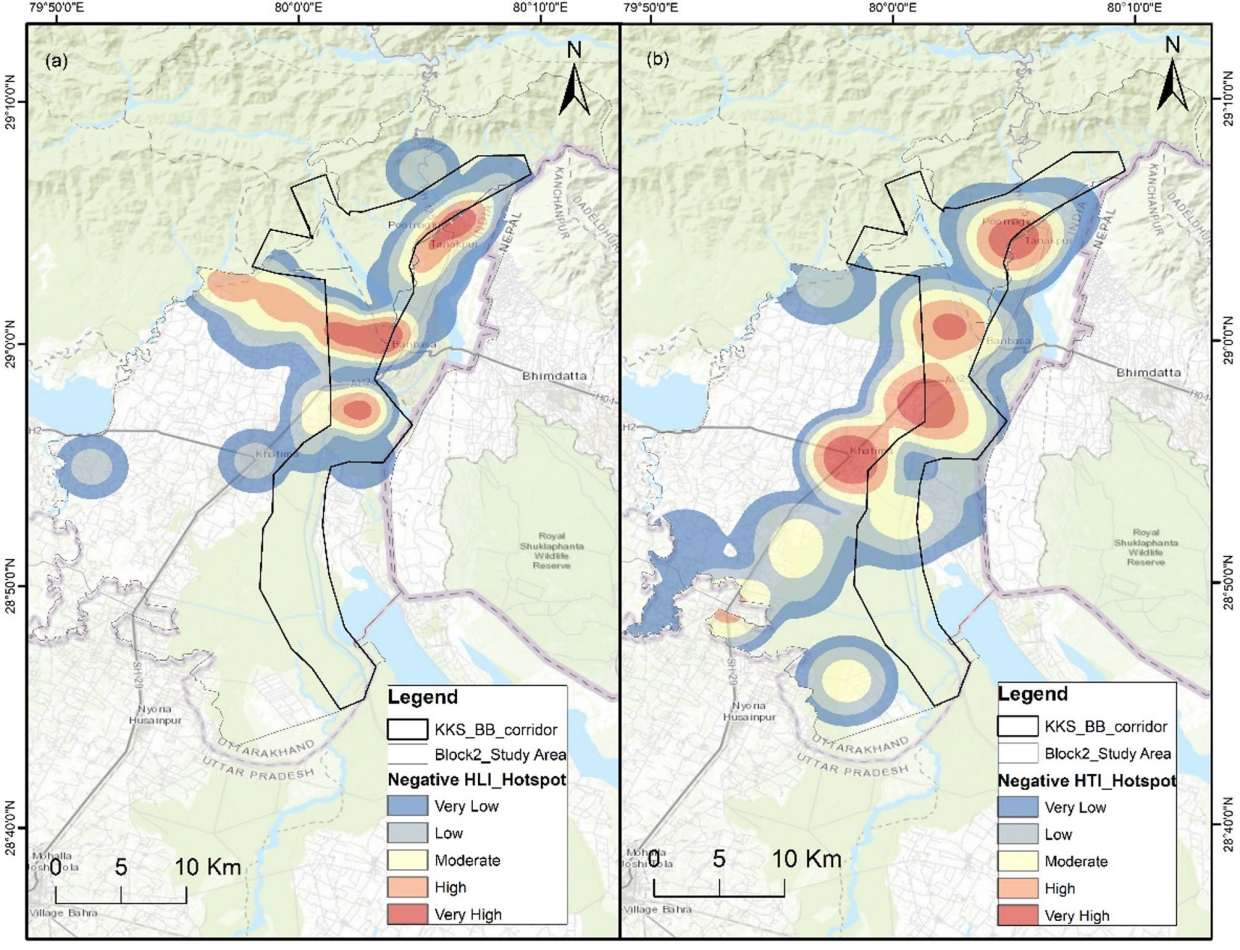
Spatial risk hotspot map of Study Block 2, which is composed of the KKS and BB Corridor. (**a**) Negative Human-Leopard Interaction (HLI) Hotspots (**b**) Negative Human-Tiger Interaction (HTI) Hotspots. (KKS: Kilpura-Khatima-Surai, and BB: Boom-Brahmadev) The map was generated in ArcGIS Desktop 10.7. 0 (Esri, Inc.) software and used India Topographic map as basemap; Service Layer Credits: Sources: Esri, HERE, Garmin, Intermap, increment P Corp., GEBCO, USGS, FAO, NPS, NRCAN, GeoBase, IGN, Kadaster NL, Ordnance Survey, Esri Japan, METI, Esri China (Hong Kong), © OpenStreetMap contributors, and the GIS User Community.

For spatial risk mapping, we had 125 input geocoordinate locations of negative HLCI incidents in Block 1 and 97 locations in Block 2. The number of incidents in these locations significantly differed for the tigers and leopards in each study block (Block 1 *p* = 2.27E-05; Block 2 *p* = 2.66E-07). The density of negative HLCI events in the Kernel Density Estimation (KDE) hotspot map of Block 1 ranged from 0 to 61.6, and that in Block 2 ranged from 0 to 6.6 (Fig. 1). In Block 1, the KDE values of leopard-related incidents range from 0 to 4.9, and those of tiger-related incidents range from 0 to 22.8. In Block 2, the leopard KDE ranges from 0 to 2.5, and tiger incidents range from 0 to 0.4. The magnitude of the tiger-related negative HWI is substantially low in Block 2. However, the spatial spread of tiger-related negative interaction priority risk areas was greater than that of leopard-related priority risk areas in both study blocks (Figs. 2 and 3). The high-risk spatial zones for leopard-related negative interaction are restricted to the eastern flank of Block 1 in human-dominated areas away from the Kosi corridor (Fig. 2). The tiger high-risk zone covers both the Kosi corridor and the eastern flank of study block 1. In Block 2, the leopard high-risk zone is restricted to the Khatima–Kilpura segment of the Kilpura-Khatima-Surai (KKS) corridor and Boom-Brahmadev (BB) corridor areas (Fig. 3). The number of tiger incidents is significantly greater than the number of leopard incidents in Block 1. Hence, the overall negative HLCI hotspot map resembles the tiger hotspot map. However, in Block 2, the negative HLCI hotspot map is more similar to the leopard hotspot map because it has a greater number of incidents than the tigers.

### LULC classification

**Fig. 4:**
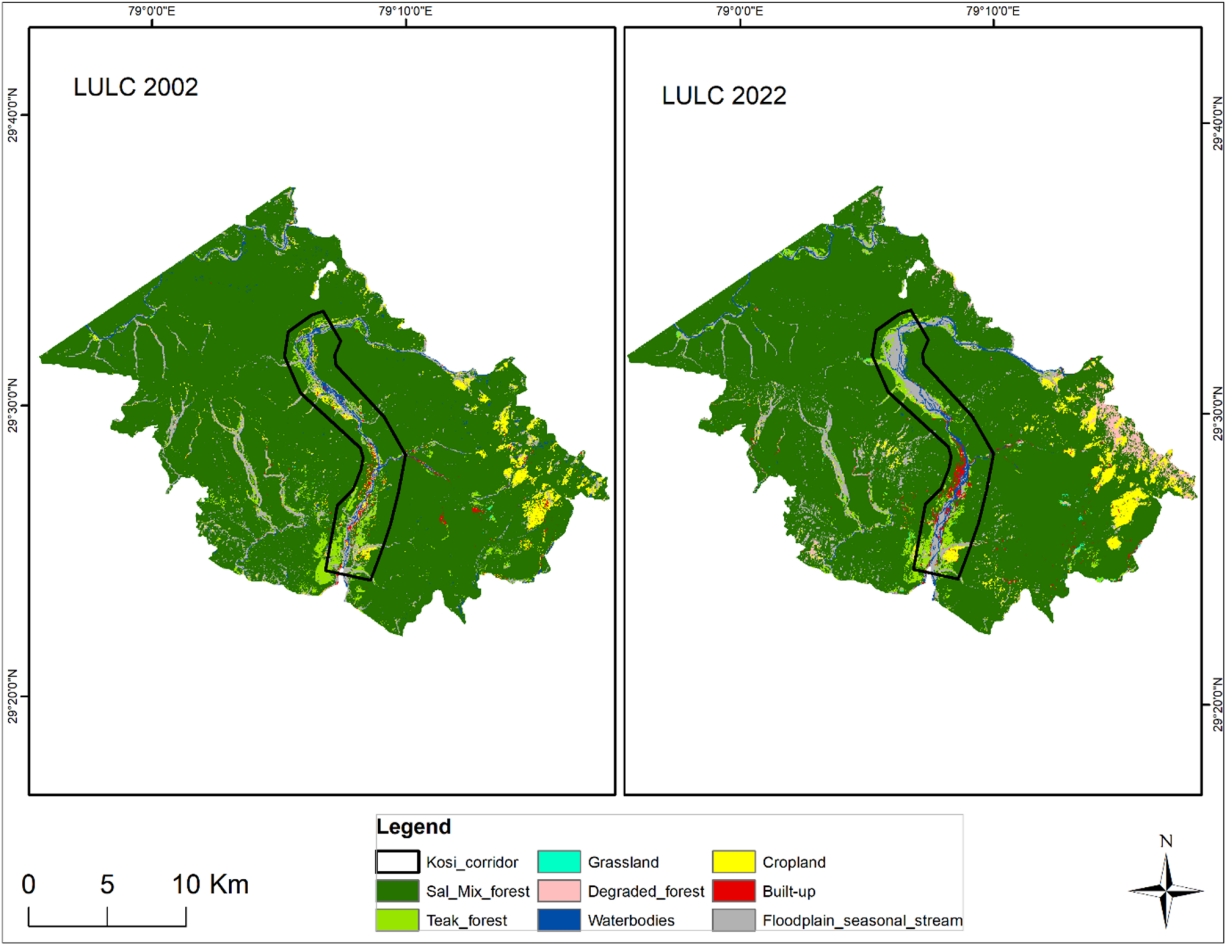
Land-use land-cover (LULC) maps of 2002 and 2022 in study block 1 with Kosi corridors. The map was generated in ArcGIS Desktop 10.7. 0 (Esri, Inc.) software using freely available satellite images of Landsat 7 ETM c2 level-2 and Landsat 9 OLI/TIRS c2 level-2 from U. S. Geological Survey (USGS EarthExplorer, https://earthexplorer.usgs.gov/).

**Fig. 5:**
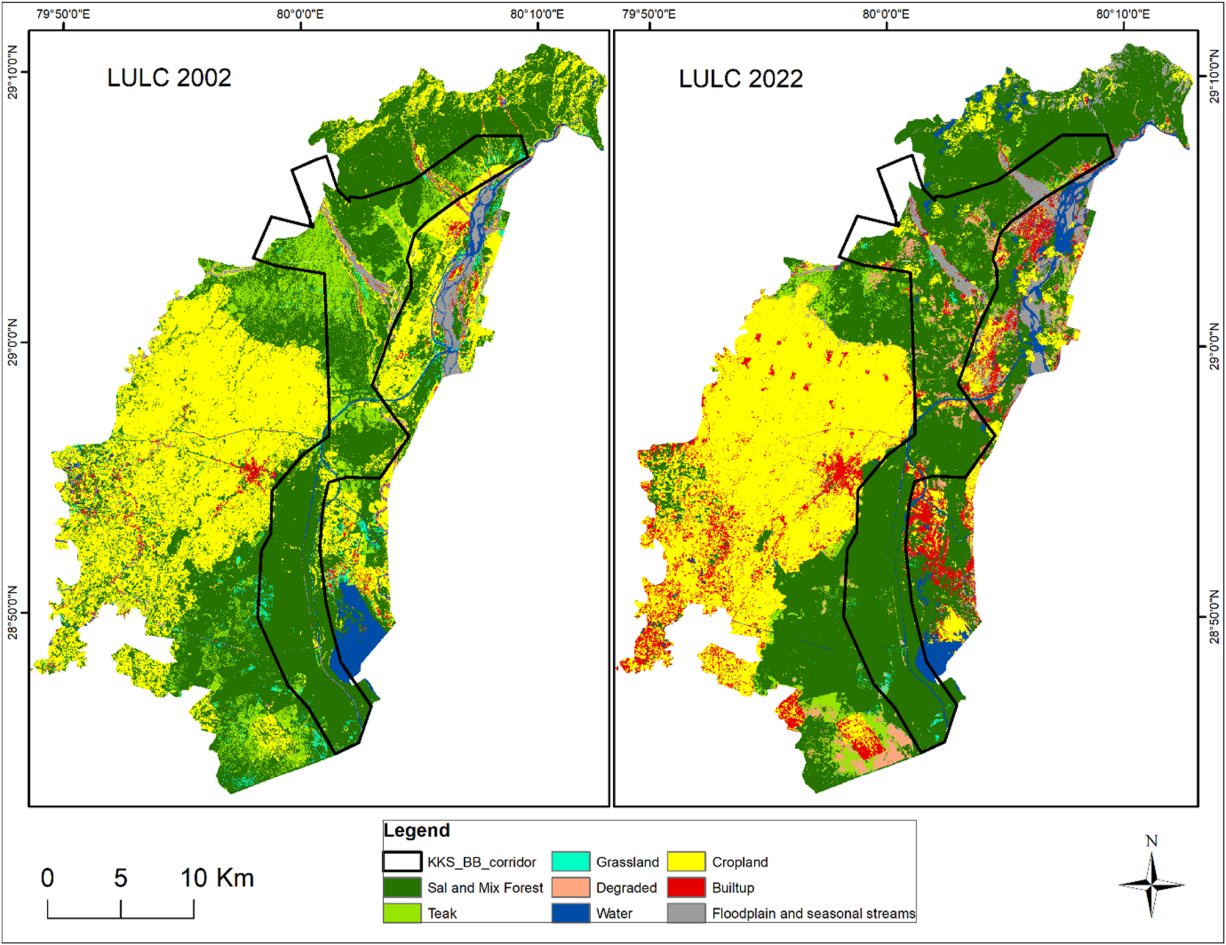
Land-use land-cover (LULC) maps from 2002 and 2022 of study block 2 with the Kilpura-Khatima-Surai (KKS) and Boom-Brahmadev (BB) corridors. The map was generated in ArcGIS Desktop 10.7. 0 (Esri, Inc.) software using freely available satellite images of Landsat 7 ETM c2 level-2 and Landsat 9 OLI/TIRS c2 level-2 from U. S. Geological Survey (USGS EarthExplorer, https://earthexplorer.usgs.gov/).

LULC classification was performed for 2002 and 2022 to evaluate the habitat transformation in the delineated wildlife corridor and adjoining areas. Eight LULC classes were selected on the basis of their habitat preferences and influence on the wildlife movement of large carnivores. The user accuracy, producer accuracy, overall accuracy, and kappa coefficient for LULC classification of blocks 1 and 2 for 2022 are 81% (Supplementary Tables S1 and S2). The dominant class is the Sal_Mix forest in the landscape, which includes all types of woodlands other than teak forests. In Block 1, the sal_mix forest class has decreased since 2002, whereas the other categories have increased, with the highest increase in the degraded forest of approximately eight sq. km from 2002 to 2022 (Supplementary Table S3, Fig. 4). However, in Block 2, the sal_mix forest, degraded forest, water body, built-up, floodplain and seasonal stream classes increased from the 2002, with a significant increase in the built-up area of approximately 50 sq. km. The teak forest, grassland, and cropland classes have decreased, with the greatest decrease in the teak forest area of approx. 41 sq. km in Block 2 (Supplementary Table S4, Fig. 5) which may be attributed to the teak removal policy of the government. The LULC class change detection results between 2002 and 2022 in blocks 1 and 2 are given in Supplementary Tables S5 and S6, respectively.

### Enhanced vegetation index (EVI) change

**Fig. 6:**
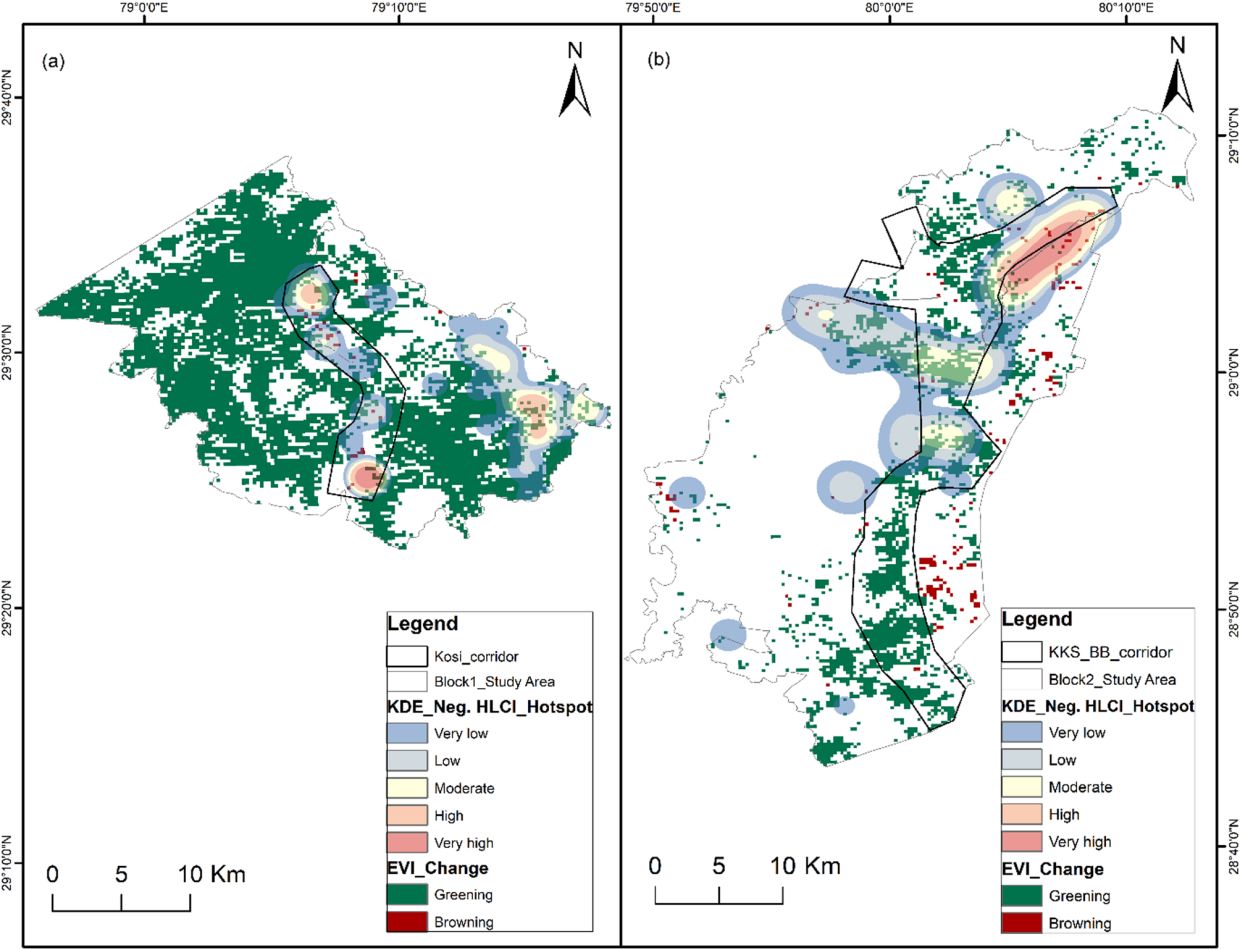
Enhanced vegetation index (EVI) changes in the study area showing the greening and browning of vegetation over two decades between 2002 and 2022 superimposed with spatial risk zones (Neg. HLCIs) and delineated wildlife corridors. (**a**) Study block 1 with the Kosi corridor (**b**) Study block 2 with the Kilpura-Khatima-Surai and Boom-Brahmadev corridors. The map was generated in ArcGIS Desktop 10.7. 0 (Esri, Inc.) software using freely available MODIS global 250 m data EVI layer (MOD13Q1 V6.1) from U.S. Geological Survey (USGS), and National Aeronautics and Space Administration (NASA) Earth Observing System Data and Information System EOSDIS (https://lpdaac.usgs.gov/products/mod13q1v061/).

The change in the EVI highlights the vegetation quality transformation from 2002 to 2022. The EVI change ranged from − 0.21 to 0.35 in Block 1 and from − 0.22 to 0.3 in Block 2. The EVI change observed in the study area over the twenty-year period was low. Overlaying the KDE negative HLCI hotspot map over the vegetation transformation map shows presence of browning areas, i.e., degradation in vegetation quality in the corridors mostly in the high-risk zones (Fig. 6).

### Night-time light (NTL) change

**Fig. 7:**
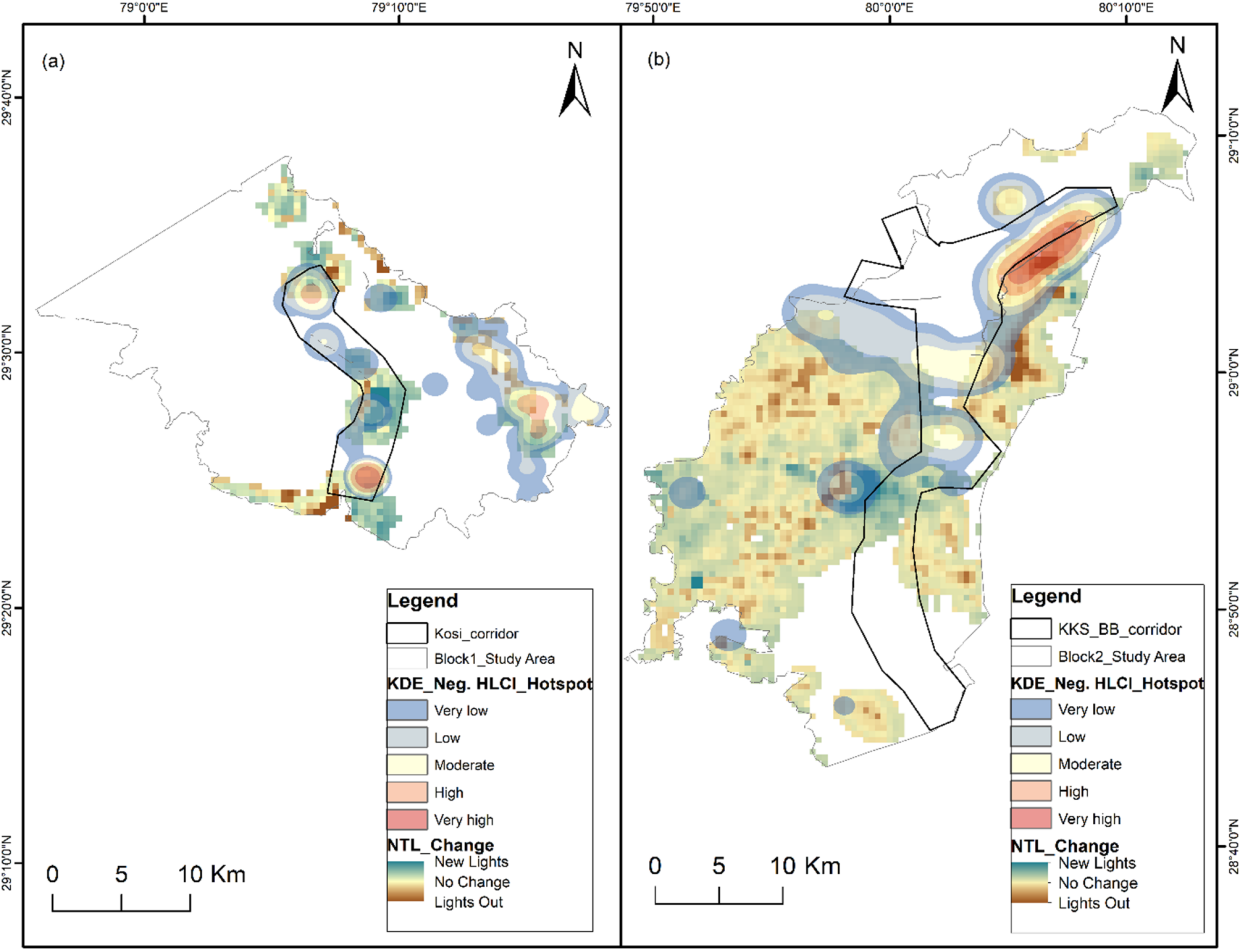
Night-time light (NTL) decadal change between 2012 and 2022 in the study area showing the expansion of human activity centres with overlayed spatial risk zone maps of negative HLCI for spatial comparison. (**a**) Study Block 1 (**b**) Study Block 2. The map was generated in ArcGIS Desktop 10.7. 0 (Esri, Inc.) software using freely available annual VIIRS Night-time Lights (VNL) V2.2 and V2.1 averaged masked data of Earth Observation Group (EOG) from Colorado School of Mines (https://eogdata.mines.edu/products/vnl/).

The change in NTL from 2012 to 2022 shows an increase in light emissions near the high- and very high-risk negative HLCI zones (Fig. 7). The difference in NTL in Block 1 ranges from − 3.71 to 5.44, and that in Block 2 ranges from − 5.11 to 5.51 between 2012 and 2022. The decadal change in NTL in the study area is moderate. An increase in NTL indicates the expansion and growth of human settlements, activities and development. The increase in night light i.e., increased human activity affects the movement path of wildlife, further squeezing the bottleneck areas. Study block 1 shows increased light, i.e., new lights around the corridors. Study block 2 shows a high increase in light around the Khatima range, forming the bottleneck of the KKS corridor and areas along the Sharda river.

### Cattle distribution in negative HLCI hotspots

**Fig. 8:**
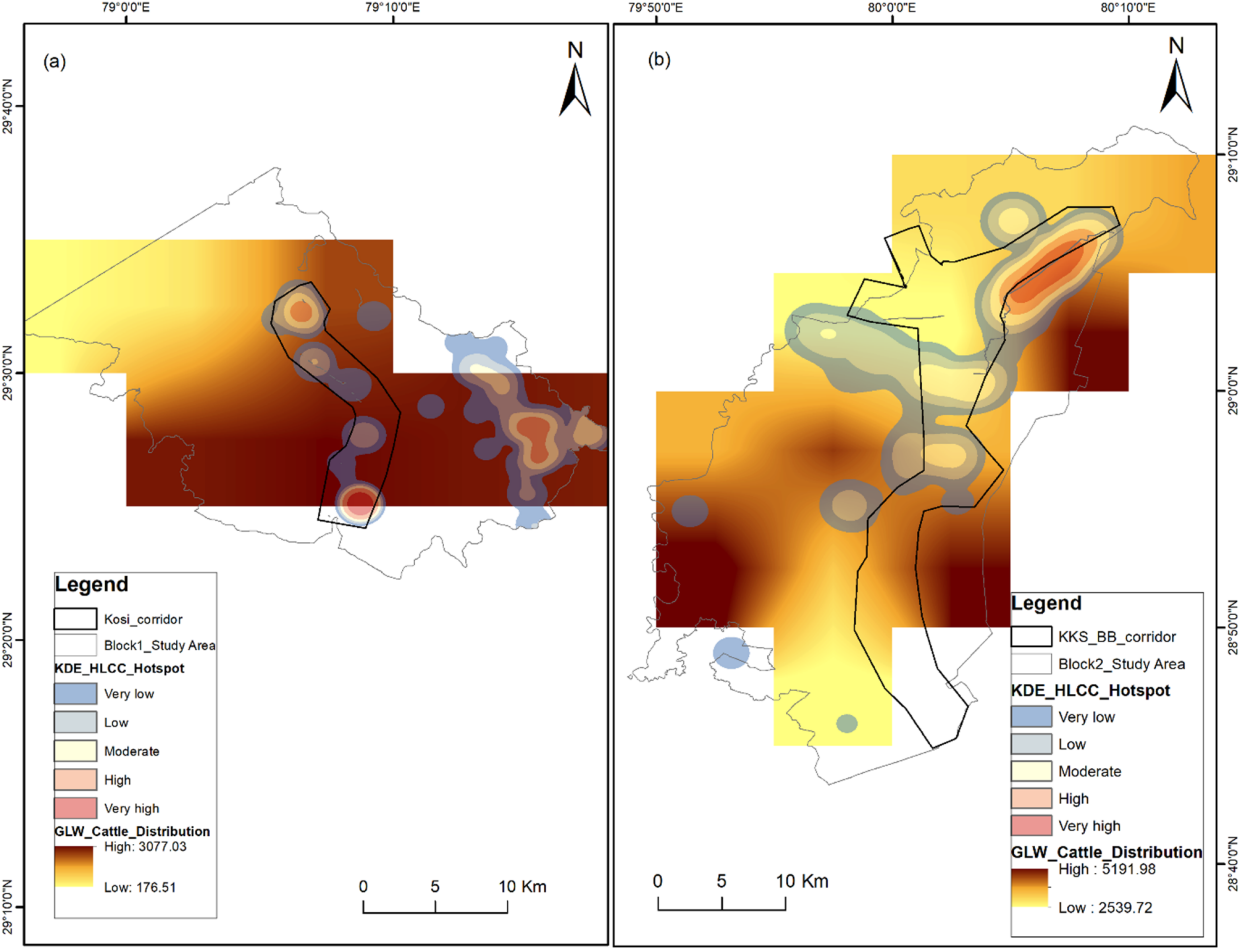
Map showing livestock (cattle) density around the wildlife corridors superimposed with the spatial risk hotspot map of the negative HLCI in the study area. (**a**) Study block 1 with the Kosi corridor. (**b**) Study block 2 with the Kilpura-Khatima-Surai and Boom-Brahmadev corridors. The map was generated in ArcGIS Desktop 10.7. 0 (Esri, Inc.) software using freely available cattle densities (dasymetric) from the FAO’s Gridded Livestock of the World (GLW) v4 map layer (https://dataverse.harvard.edu/dataverse/glw_4).

We extracted the study area mask from Gridded Livestock of the World (GLW) v4 with the dasymetric method (DA) for the cattle distribution map. The cattle density (animals per sq. km) in the Block 1 area ranges from 176.51 to 3077.03 and 2539.72–5191.98 in the study Block 2 (Fig. 8). Spatially, the high-risk zones of negative HLCI overlaps with the high cattle density in adjoining areas. However, there is substantial difference in the cattle density between the study blocks.

### Impact analysis

The models with both habitat and sympatric species effects had better goodness-of-fit with a lower AICc than those without sympatric species effects because the influence of species-specific patterns plays an important role in shaping spatial HLCI patterns (Table S7). The GLM in study block 1 for leopards revealed a significant (*p* < 0.05) effect of anthropogenic activity presence (NTL change) and the negative human-tiger interaction (HTI) hotspot on the negative human-leopard interaction (HLI) hotspot (Table S8). The decrease in night-time light had a significant negative effect on negative HLI, indicating that the less or no anthropogenic settlements and disturbances in forested habitats have a diminishing effect on leopard related negative HWI (B = −1.266, Table S9). The HTI hotspot had a significant positive effect on HLI (B = 0.114, Table S9) signifying common areas where both sympatric large carnivores have negative HWI (Fig. 2). The tiger-related negative interaction model in study block 1 revealed a significant effect of vegetation health (EVI change), LULC change and HLI (Table S8). Greening has a significant negative effect on negative HTI, indicating a decrease in tiger-related negative interaction with improved vegetation quality (B = −0.428, Table S10). The LULC change (B = 0.012) and negative HLI hotspot (B = 0.871) had a significant positive effect on the tiger negative interaction sites; i.e., the land-use change had an incremental effect on the negative HTI hotspots (Table S10) and common hotspot areas for both sympatric species (Fig. 2). For both the tiger and leopard combined (HLCI) hotspots, the influence of cattle density was significant, with a negligible effect (Table S11). The tiger and leopard negative interaction hotspot significantly affects the overall negative HLCI hotspot (Table S11), highlighting the importance of the influence of the species-level hotspot pattern on the overall large carnivore conflict hotspot pattern.

In study block 2, the GLM for leopards revealed a significant effect of anthropogenic presence (NTL change) and negative HTI (Table S8), similar to the trends in study block 1. The decrease in night-time light, symbolizing the decrease in anthropogenic activities, has a significant negative effect on HLI, i.e., a diminishing effect on the negative HWI with leopards (B = −1.119, Table S12). The negative HTI hotspot has a significant positive effect on the negative HLI hotspot (Table S12). The model for the tiger in study block 2 revealed a significant positive effect of the decrease in night-time light, indicating higher negative HTI in areas with lower anthropogenic activity (B = 0.569, Table S13). The negative HLI hotspot also has a significant positive effect on negative HTI in study block 2 (B = 0.668). The cattle density also had a significant negligible effect on HTI (Table S13). The model for the overall negative HLCI (combined tiger and leopard) in study block 2 revealed a significant effect of vegetation quality improvement (Greening), HLI and cattle density (Tables S8 & S14). The improvement in vegetation quality (Greening) has a significant negative effect on the negative HLCI (B= −0.441), indicating that healthy vegetation areas have a low negative HLCI intensity. HLI had a significant positive effect on HLCI (B = 2.412), whereas the effect of HTI was not significant for HLCI in study block 2. The effect of cattle density on negative HLCI was significant but negligible (Table S14).

## Discussion

Our results highlight the significant implications of habitat transformations for large carnivore spatial hotspots of negative HWI, which also vary with species and region. The findings support our hypotheses and assumptions highlighting the habitat transformations over decades is one of the significant threats to human-wildlife co-existence and critical drivers for negative interaction. The effects of habitat change from natural habitats to human-modified landscapes or degraded habitat quality on tiger and leopard negative HWI hotspots varied across the study blocks. The habitat implications for negative HLI hotspots show a similar trend in both study blocks, where the negative HLI is lower in areas where anthropogenic activities have decreased from the past (decrease in NTL change), highlighting that a decrease in anthropogenic disturbance can improve co-existence (Tables S9 & S12). An earlier study on leopards also revealed that animals residing in sparsely populated areas tend to avoid proximity to human settlements, whereas animals living in densely populated areas are adapted to living in close proximity with humans and frequently manoeuvre through the settlements for food at night (temporal partitioning), avoiding encounters with humans^25^. The sparse human settlements in wildlife corridor areas provide better permeability for safe wildlife movement, which is critical for minimizing the negative HWI^26^. The significant positive effect of HTI hotspots on HLI hotspots and vice versa in both study blocks infers spatial overlap and common critical (high-risk zones) negative HWI hotspot areas of these two sympatric species in the landscape, which is also clearly visible in the species hotspot map (Figs. 2 and 3).

Our findings show that vegetation quality is crucial for minimizing the negative HWI, as highlighted by the model for negative HTI in study block 1, which indicates that improvement in vegetation health (Greening) has a diminishing effect on tiger-related negative HWI hotspots (Table S10). This model also reveals the importance of land-use changes, which have an incremental effect on the negative HWI, possibly because of rapid development (urbanisation) to which species cannot adapt, and reduced habitat permeability. Human-modified habitats like croplands are still permeable for large carnivores; however, their conversion to built-up areas may increase the barrier effect, limiting the permeability of the corridor. Land use changes affect habitat quality, which cascades over the natural prey base of large carnivores^27–29^. The growth of hotel and resort businesses, increasing night traffic, and human presence in Kosi corridor areas in the last decade has been rapid, substantially reducing the permeability of the corridor.

In study block 2, the model reveals that the negative HTI increases in areas where anthropogenic activity (NTL change) has decreased (Table S13), which may be attributed to the location of tiger-related incidents, mainly inside the forest areas. Most of the negative HWI incidents with tigers in the KKS and BB corridor areas have been reported from forested habitats while grazing or fuelwood collection (Ranjan, Unpubl.). The effect of cattle density was significant, with near-zero values in the negative HTI and HLCI models, which may have had a small-scale direct effect on the negative HWI (Tables S11, S13, & S14). However, the indirect impact of livestock through habitat degradation and resource competition with wildlife may have far-reaching implicit consequences affecting the structural and functional levels of prey-predator dynamics, with anthropogenic elements acting as a third pillar^29–32^. The improvement in vegetation health has a diminishing effect on the negative HLCI, evident in study block 2 (Table S14), as the human sprawl in this area is extensive, affecting the quality of the forest and habitat. Hence, enhancing vegetation health provides a conducive environment for elusive large carnivorous species. The demographic conditions of Block 2 differ from those of Block 1, with more dense human habitation, industries, and high anthropogenic pressure on forest-like grazing and non-timber forest produce (NTFP) collection.

The models for which the effects of habitat changes—such as LULC changes, increase in human activity (increased NTL), and deterioration of vegetation health (browning)—are not statistically significant do not necessarily mean that they do not influence the HWI. The impact of habitat changes on wildlife is not always direct and perceivable over a short duration after the changes have occurred^33,34^. However, over a longer period of time, the effects of habitat changes can be observed as highlighted in our study, which analyses decadal variations in habitat attributes. Studies have attempted to establish the relationships between HWC and habitat characteristics, socioecological factors, and anthropogenic disturbances, which are not always strongly or linearly correlated^13,20,35–37^. Thus, our approach with GLM to understand the impacts of habitat changes highlights crucial factors that have significant effects. However, we may not say that the habitat attributes which are not statistically significant do not have any implications on the negative HWI. The relationship of a species to its habitat in a natural ecosystem is complex, direct, indirect, nonlinear, and multifaceted. This study also highlights the importance of considering the role of sympatric species while understanding and determining the underlying factors and patterns of HWI, as all the models show better fitness and significant effects of sympatric large carnivore species.

Our study revealed that the negative HLCI in the eastern terai landscape of Uttarakhand is mainly due to tigers and leopards, which constitute the major share of livestock depredation. Human casualties are chiefly related to tigers, with most incidents occurring in forest areas during NTFP collections or due to sudden encounters. The greater number of tiger incidents in Block 1 can be attributed to the high tiger density in the Corbett Tiger Reserve (CTR) and adjoining areas^10,38^. The tiger density in the CTR is the highest in India among all PAs^10^. The tiger density in study block 2 is lower than that in block 1; however, the density has recently increased^10^. The buffer areas of NWLS and PTR around the KKS and BB corridors are dotted with human settlements, both legal and encroachment, and are frequently fragmented due to anthropogenic disturbances, which may be crucial factors for higher human casualties in Block 2 than in Block 1. The greater number of human casualties related to tigers than to leopards may also be attributed to the different behavioural attributes of these two sympatric predators^39,40^. In general, leopards avoid direct human confrontation^25,39^. Leopards are more adaptable and manoeuvre human-dominated landscapes conveniently than tigers^41,42^. The increased anthropogenic disturbances in the natural ecosystem of wildlife also affect their behaviour and nature in response to human presence^43,44^.

Communities living around forest areas depend on forest resources for fuelwood, fodder, livestock grazing, and other domestic needs of the household, where socioeconomic conditions are not good^45–47^. The increasing human population and expansion have led to habitat encroachment, which is sometimes favoured by political scenarios^37^as in the case of our study area. Livestock herding and rearing are primarily dependent on forest resources, and few pastoralist communities in our landscape, such as *Van Gujjars*, depend entirely on forest fodder for their livestock, and *Bakrawal* (sheep and mountain goat herders) in the winter season move to lower elevations in the Shivalik and Terai. This significantly increases foraging competition between wild ungulates and livestock, restricting the availability of natural forage for wildlife species^48^. It also affects prey base availability and the preference of available prey for top predators such as tigers and leopards^49,50^. These anthropogenic interferences and disturbances are very high outside PAs and affect the habitat use patterns of wildlife species^31,32^. The increased livestock pressure decreases the availability of foraging pasture grounds for wild prey populations in forest habitats, forcing prey species to move towards agricultural fields and villages for food^25,39,51^.

This study revealed land-use changes over two decades, and we observed low to moderate changes in vegetation cover and LULC patterns in our study blocks. The LULC change highlights the significant transformation of sal and mix forest to degraded forest in block 1. The degradation of habitat quality is caused mainly by erosion, invasive plant species such as *Lantana camara* and *Ageratina adenophora*, and vegetation loss due to anthropogenic disturbances, which are significant problems for the sanctity of wildlife habitats^19,52^. The diversity of habitats is favourable for wildlife species, especially large mammals such as tigers and leopards, which also provide resilience to the ecosystem to sudden changes^49,53^. However, the increasing human population and rapid change in LULC patterns in the terai landscape are concerns about wildlife conservation outside PAs in a human-dominated mosaic landscape. Land-use changes, degrading vegetation quality or health, and growth in human footprints negatively affect the wildlife corridor and movement, acting as stressors for wildlife species^2,54^. The abundant livestock around wildlife habitats and corridors also induces behavioural plasticity in large carnivores for easy livestock prey, especially in areas with low natural prey bases^49^. The increasing human-induced pressure on wildlife habitats and corridors has a funnelling effect, creating tight bottlenecks^55,56^. It affects the permeability of corridors and hinders free and safe wildlife movements.

## Conclusion

Our research focuses on the issues and spatial factors responsible for negative human–large carnivore interactions outside PAs in the wildlife corridor habitats of human-dominated landscapes. Our study emphasises on the value of wildlife corridors for human-wildlife co-existence, while also drawing attention to the risks posed by urbanisation and human expansion in these areas. This study highlights that the implications of habitat changes are significant for HLCIs, leading to conflict, and the role of sympatric species in shaping these patterns. The study reveals that the NTL change can be an important proxy to determine the influence of anthropogenic activities on HWI, given the availability of long-term data. The implications of habitat changes vary with landscape and regional attributes, as observed in our study at two sites with varied underlying factors other than habitats. Landscapes are rich in biological diversity and human populations, which present a challenging scenario for long-term conservation and co-existence. Understanding the effects of habitat changes on negative HWI spatial patterns for particular landscapes is essential for effective planning and management interventions for minimizing HWC. As many of these risk zones of negative HWI may also witness conflict requiring urgent conservation attention. The efforts of governments, organizations, researchers, institutions, and conservationists have yielded positive results for wildlife protection and conservation in India, with an increase in the population of tigers and co-predators such as leopards. However, it has also brought a more significant challenge of accommodating conservation needs with human needs in a sustainable way for the peaceful co-existence of man and the species.

Using wildlife attack data for this study provides a better and more empirical understanding of HWC issues at local and regional scales. However, the lack of a precise location of incidence affects the accuracy of analysis and hotspot identification. The lack of data regarding retaliatory actions towards wildlife also affects the precision of risk zones in truest sense. This study extends beyond statistical methods to explore and examine the factors that play a vital role in the negative HWI. This highlights the significance of wildlife corridors and their ecological sanctity for long-term conservation, as well as providing legal protection status to these corridors. Based on this study, maintaining the balance between development and habitat protection should be our utmost priority to reduce negative interactions between humans and large carnivores.

## Methods

### Study area

**Fig. 9:**
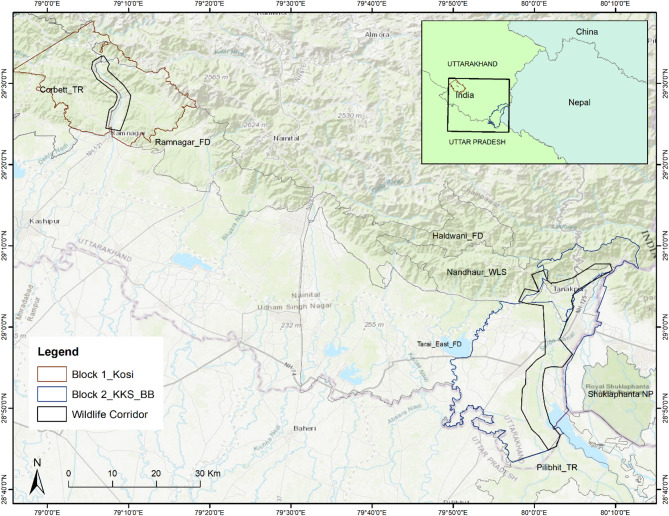
Study area map showing the two study blocks and the focal wildlife corridors i.e., Kosi, KKS and BB in the Terai arc landscape in Uttarakhand east of the Corbett Tiger Reserve to the Indo-Nepal border. (KKS: Kilpura-Khatima-Surai, and BB: Boom-Brahmadev). The map was generated in ArcGIS Desktop 10.7.0 (Esri, Inc.) software and used India Topographic map as basemap; Service Layer Credits: Sources: Esri, HERE, Garmin, Intermap, increment P Corp., GEBCO, USGS, FAO, NPS, NRCAN, GeoBase, IGN, Kadaster NL, Ordnance Survey, Esri Japan, METI, Esri China (Hong Kong), © OpenStreetMap contributors, and the GIS User Community.

The study area consists of wildlife corridors for large mammals and adjoining forests in the Terai Bhabhar landscape, which are part of the broader Shivalik corridors^10,57,58^. It lies in the Terai Arc Landscape (TAL)^59^east of the Corbett Tiger Reserve (CTR) and west of the Indo-Nepal border in Uttarakhand, India. It is also part of the tiger conservation landscape (TCL) Tx2 (TCL_ID: 44)^60,61^ and tiger habitat block (THB) II and III^62^. Three wildlife corridors, i.e., the Kosi, Kilpura-Khatima-Surai (KKS), and Boom-Brahmadev (BB) corridors, were included in the study. The study sites are categorized into two study blocks for convenience on the basis of spatial location: Block 1—Kosi corridor; Block 2—KKS; and the BB corridor. Study block 1 covers a geographical area of 250 sq. km spanning from 79.0925 N, 29.6024 E to 79.1726 N, 29.3741 E in north-south extent (Fig. 9). Study block 2 covers an area of 700 sq. km. spreading north-south from 80.1907 N, 29.1822 E to 79.9571 N, 28.7227 E (Fig. 9).

The Kosi corridor connects the CTR with the Ramnagar Forest Division (FD) and Pawalgarh Conservation Reserve along the Kosi River east of the CTR in Uttarakhand. The KKS corridor connects the Nandhaur Wildlife Sanctuary (NWLS) in Uttarakhand with the Pilibhit Tiger Reserve (PTR) in Uttar Pradesh and the Indo-Nepal border in the Khatima forest range. The BB corridor connects NWLS to the Kanchanagar FD in Nepal, a transboundary landscape that expands to Sukhlaphanta National Park in Nepal^10,59^. KKS and BB corridors have interconnected habitats and are thus considered together as Block 2 for this study. The Kosi corridor comprises parts of the Kosi and Kota forest ranges in the Ramnagar FD; the Bijrani, Sarpduli, and Mandal forest ranges in the CTR; and the Mohan range in the Almora FD. The KKS corridor consists of the Kilpura, Khatima, and Surai forest ranges of the Terai East FD, Uttarakhand. The BB corridor consists of the Sharda forest range of the Haldwani FD, parts of the Kilpura forest range, and the Dogadi and Boom forest ranges of the Champawat FD in Uttarakhand, India.

The major forest types are moist and dry deciduous forests interspersed with alluvial and riverine grasslands^63,64^. Sal forests, teak forests, and mixed forests are primarily found in landscapes comprising tree species such as *Terminalia alata*,* Anogeissus latifolia*,* Lagerstroemia parviflora*,* Holoptelia integrifolia*,* Ehretia laevis* and *Aegle marmelos*^65^. The crucial large mammal species in the region are Asiatic Elephant (*Elephas maximus)*,* Panthera tigris*,* Panthera pardus*, Sloth Bear *(Melursus ursinus)*, Sambar deer (*Rusa unicolor)*, Spotted deer (*Axis axis)*, and Northern Red Muntjac (*Muntiacus muntjac*). The study area includes the 2B Himalaya-West Himalaya and 7 A Gangetic Plain-Upper Gangetic Plain biogeographic provinces of India^66^. The landscape is also a densely populated area with rapid growth and urbanization. The natural vegetation is interspersed with agricultural land and human settlements.

### Data collection

The data were collected during the fieldwork under the project funded by the National Mission on Himalayan Studies. We collected ex-gratia payment records for HWC between 2006 and 2020 from the field offices of the Uttarakhand Forest Department (UKFD), with data gaps between 2006 and 2009 at some locations of block 1. The data formats and details were inconsistent across all the documents; thus, we had to scrutinize and reorganize the data as per the requirements of the study. Hence, we have used the data from 2009 onwards for block 1 and from 2006 onwards for study block 2. We did not use a few incident reports that did not mention the date of the event. The record registers contained information such as the incident date, the species involved, the number and type of livestock killed, the human casualty/death, the victim’s name or name of the livestock owner, and the village/locality/forest beat or compartment where the incident occurred (in cases where the incident occurred in forest-village fringe areas, only the name of the village was mentioned). The incidents reporting the forest beats were considered as events occurring inside the forest in that particular forest beat, whereas incidents in the village areas or crop fields, or on the forest-village fringes that do not mention forest beat were considered for that village/hamlet. All the records of livestock depredation lacking species name involved in the incident, were marked for large carnivore. The records mentioning the species name were sorted under tiger or leopard-specific events. We cross-checked the records with forest officials and community members during household surveys. We filtered the data related to large carnivores (tiger and leopard) from the ex-gratia records.

### Spatial risk mapping

The number of incidents related to the tigers and leopards at each location in each study block was statistically compared via a two-tailed paired t-test. This study uses geostatistical techniques to analyze point data in terms of spatial statistics and spatial analysis for the risk assessment of their spatial patterns. The geolocations with the number of incidents associated with each village or beat were analysed using spatial autocorrelation (Moran’s I) in Arc GIS 10.7, which was not significant in both the study blocks for Tiger and Leopard (p-value > 0.05), thus, we used kernel density estimation (KDE).

Kernel density is an effective GIS analysis that helps identify event hotspots^67,68^. We used the KDE technique in ArcGIS 10.7 to map the spatial negative HLCI hotspot based on the intensity and magnitude of the incidence in an area or village^68,69^. The bandwidth was set to default, which computes specifically to the input dataset using a spatial variant of Silverman’s Rule of Thumb that is robust to spatial outliers. The default search radius for all the hotspot maps was also compared with the different bandwidths of 1 and 2 km maps, where the default bandwidth map showed better zonation and spread as per the field conditions. We populated the field with the number of incidents at each location. The geocoordinates of the location of incidence were not available for most of the cases; thus, we used one central geolocation for each village or forest compartment/beat. This may cause spatial biasedness from the actual site of incidence because it will show more density closer to the location. However, when we try to assess the risk zones or areas with higher chances of negative interaction, this technique is empirical and better suited for hotspot identification, as the areas closer to the villages will have a higher probability of negative HWI. The kernel density highlights the area of high intensity and magnitude of the negative HLCI, where multiple incidents near any village or forest beat areas are recurring. The kernel is an estimator that functions by generalizing or smoothing discrete point data into a continuous surface area^70,71^. The KDE hotspot map was reclassified into five categories, namely, very low-, low-, moderate-, high-, and very high-risk zones, on the basis of the magnitude and intensity of negative HLCI incidents (Fig. 1). The areas with null values have not been mapped in the spatial risk zone maps.

### LULC

Analyzing changes in LULC is essential for examining urban development worldwide^72^. LULC maps for 2002 and 2022 were prepared via Landsat 7 and 9 satellite images acquired from the USGS Earth Explorer to assess the changes over two decades. We selected 2002 as a reference year because the state of Uttarakhand was formed in 2000, significantly impacting the study area’s developmental activities and demography. We considered the satellite images of May for both years when the majority of the croplands were harvested. The lack of vegetation in croplands makes distinguishing between grasslands, croplands, and degraded invasive species forests convenient^73–75^and the cloud cover is minimal during this period. The Landsat 9 OLI/TIRS level 2 product for 2022 and the Landsat 7 ETM level 2 product for 2002, WRS_PATH = 145, WRS_ROW = 40, were used with less than 10% cloud cover. LULC classification was performed via the supervised maximum likelihood classification tool in ArcGIS 10.7. The classification was assessed for accuracy on the basis of the kappa coefficient (κ) using random accuracy points^76,77^. We used field data of the vegetation plots and habitat types to ensure the accuracy of the training samples. A minimum of 50 data point locations of each class type was used in the training sample, with more data points for widespread classes proportional to their coverage.

The study area was classified into eight LULC classes: 1) Sal_Mix forest, which is composed of sal, mix, and other broadleaf forests except Teak; (2) Teak forest; (3) Grassland, which is inclusive of riverine grasslands; (4) Degraded forest, which includes barren forestland due to landslides or erosion or any natural hazards, with areas under invasive species such as lantana, and forest areas disturbed due to anthropogenic activities in the past; (5) Water bodies, which include all natural and artificial water bodies and canals; (6) Cropland; (7) Built-up; and (8) Floodplain and seasonal streams, which include monsoon streams, river banks, and dry riverbeds. The classes are based on the habitat type use and preference of large carnivores^20,78,79^.

### Change analysis

The transformation of habitat characteristics and composition significantly affects wildlife behaviour and activity^18,19^. All the change analyses were performed in ArcGIS 10.7. We examined the changes in LULC, enhanced vegetation index (EVI), and night-time light (NTL) in the wildlife corridor and its adjoining areas. Change detection in LULC was assessed between 2002 and 2022 over twenty years. The LULC map was downsampled for uniformity of resolution with MODIS data using the Resample tool in Arc GIS 10.7. The change in the EVI was also examined between 2002 and 2022 via MODIS global 250 m data (MOD13Q1 V6.1). The EVI minimizes canopy–soil variations and improves sensitivity over dense vegetation conditions. It is crucial to assess changes in corridor habitats, such as greening (improvement in vegetation health) or browning (degradation of healthy vegetation)^80^. The EVI ranges from − 1 to + 1, and for healthy vegetation, it ranges from 0.2 to 0.8.

The NTL change analysis was performed between 2012 and 2022 due to the limited availability of NTL data. The NTL change may or may not show a higher or stronger effect on negative HWI in one decade, but it will highlight its importance as a proxy for modelling anthropogenic impacts. We used the annual VIIRS Night-time Lights (VNL) V2.2 for 2022 and V2.1 for 2012, averaged masked for change analysis^81^. NTL data have been used as a proxy measure for urbanization, human settlement, density, and economic growth^82,83^. NTL data records nocturnal emissions of human activities and can be used to measure the urban spatial extent and changes over time^84^.

### Impact analysis

The negative HLCI hotspot map was overlaid and superimposed over the change analysis maps to understand the underlying changes in areas with high negative HWI incident frequencies and spatial associations. Using the data management tool in ArcGIS, we plotted 1000 random points in each study block. We then used the Extract Multi values to Point spatial analyst tool in ArcGIS to extract point data for each variable, i.e., EVI, NTL, LULC changes, cattle density using the GLW layer, negative HLI intensity, negative HTI intensity, and overall negative HLCI intensity using the KDE hotspot layer. All the variables were tested for multicollinearity, which showed a Variance Inflation Factor (VIF) around 1. The extracted multiple values for each point were analyzed using a generalized linear model (GLM) to examine the role and implications of EVI change, NTL change, LULC change, and cattle density on the negative HLCI, as well as the effects of sympatric large carnivorous species. It is important to assess the impacts of sympatric large carnivores on each other, as their ecology, habitat-use patterns, and conflict patterns varies with each other differently in specific landscapes^7,11,85^. The GLM was run with the gamma distribution and log link function with the KDE hotspot of a specific species (leopard, tiger, or large carnivore combined) as the dependent variable and the EVI, NTL, LULC changes, cattle density (GLW), and other sympatric species KDE hotspots as independent variables in IBM SPSS Statistics 23 software to examine their effects on the negative HLCI. The EVI change was categorized as browning (minimum to −0.1), greening (0.1 to maximum), or none (−0.1 to 0.1); NTL change was categorized as increasing (1 to maximum), decreasing (−1 to minimum), or no change (−1 to 1); and these two nominal variables were used as factors, whereas cattle density, LULC change (change matrix value) and species KDE hotspot values were used as covariates in the model. The model with the lowest corrected Akaike’s information criterion (AICc) was selected to observe the effects of different variables. The model was built with the main effects of EVI change, NTL change, LULC change, cattle density (GLW) as habitat variables, and species KDE hotspots to understand the implications of the sympatric species in tandem with the habitat parameters (Tables S7-S14).

## Supplementary Information

Below is the link to the electronic supplementary material.

Supplementary Material 1

## Data Availability

All data generated during this study are included in this published article and its supplementary information files.
