# Supplementary material for: Assessing the implications of habitat transformations on human-large carnivore interactions outside protected areas
Source: Sci Rep. 2025 Aug 6;15:28784. doi: 10.1038/s41598-025-13808-4 (PMC12328715; doi:10.1038/s41598-025-13808-4)
Supplement: Supplementary file 1 — Supplementary Material 1 [file 41598_2025_13808_MOESM1_ESM.pdf]

Assessing the Implications of Habitat Transformations on Human-Large Carnivore Interaction Outside the Protected Areas

Vivek Ranjan[1*], Ruchi Badola[1], Syed Ainul Hussain[1], Parag Madhukar Dhakate[2]

1- Wildlife Institute of India, Dehradun, Uttarakhand, India

2- Uttarakhand Forest Department, Dehradun, Uttarakhand, India

Vivek Ranjan (Corresponding Author) *
E-mail: vivek.nil@gmail.com
ORCID: 0000-0002-3796-0828

Supplementary Information

Table S 1: Confusion matrix of the 2022 LULC map of the study Block 1 showing the overall accuracy and Kappa statistics. (C_1- Sal_Mix forest, C_2 - Teak forest, C_3 - Grassland, C_4 - Degraded forest, C_5 - Waterbodies, C_6 - Cropland, C_7 - Built-up, C_8 – Floodplain and seasonal streams)

Class Value	C_1	C_2	C_3	C_4	C_5	C_6	C_7	C_8	Total	U_Accuracy
C_1	196	2	3	3	0	0	0	0	204	0.960784
C_2	0	9	1	0	0	0	0	0	10	0.9
C_3	1	2	7	0	0	0	0	0	10	0.7
C_4	1	0	0	9	0	0	0	0	10	0.9
C_5	1	0	1	0	8	0	0	0	10	0.8
C_6	0	0	0	1	0	9	0	0	10	0.9
C_7	0	0	0	0	0	0	6	4	10	0.6
C_8	1	0	1	0	0	1	1	10	14	0.714286
Total	200	13	13	13	8	10	7	14	278	0
P_Accuracy	0.965517	0.692308	0.538462	0.692308	1	0.9	0.75	0.642857	0	
Overall Accuracy	**0.913669**									
Kappa	**0.812837**									

Table S 2: Confusion matrix of the 2022 LULC map of the study Block 2 showing the overall accuracy and Kappa statistics. (C_1- Sal_Mix forest, C_2 - Teak forest, C_3 - Grassland, C_4 - Degraded forest, C_5 - Waterbodies, C_6 - Cropland, C_7 - Built-up, C_8 – Floodplain and seasonal streams)

Class Value	C_1	C_2	C_3	C_4	C_5	C_6	C_7	C_8	Total	U_Accuracy
C_1	117	5	3	2	0	3	2	1	133	0.8796992
C_2	0	12	0	2	0	1	0	0	15	0.8
C_3	2	0	8	0	0	0	0	0	10	0.8
C_4	0	0	0	10	0	0	0	0	10	1
C_5	1	0	0	0	9	0	0	0	10	0.9
C_6	4	0	0	1	2	81	3	2	93	0.8709677
C_7	0	0	0	0	0	3	18	1	22	0.8181818
C_8	0	0	0	2	0	1	0	12	15	0.8
Total	128	12	11	17	12	96	15	12	308	0
P_Accuracy	0.965517	0.692308	0.538462	0.692308	1	0.9	0.75	0.642857	0	
Overall Accuracy	**0.866883117**									
Kappa	**0.813806729**									

Table S 3: The area distribution of LULC in Block 1 in the 2002 and 2022 data sets and change over 20 years (in sq. Km and percentage)

Class Name	Area 2002 (sq. Km)	Area 2022 (sq. Km)	Area change (2002 - 2022)	Percent Change (%)
Sal_Mix forest	440.41	425.99	-14.419763	-3.27
Teak_forest	11.02	12.63	1.612992	14.64
Grassland	0.10	0.54	0.441022	462.98
Degraded_forest	0.02	8.42	8.391567	33767.52
Waterbodies	6.95	7.27	0.31905	4.59
Cropland	12.97	13.84	0.874848	6.75
Built-up	2.14	3.09	0.949291	44.34
Floodplain_seasonal_stream	25.82	27.64	1.813959	7.02

Table S 4: The area distribution of LULC in Block 2 in the 2002 and 2022 data sets and change over 20 years (in sq. Km and percentage)

Class Name	Area 2002 (sq. Km)	Area 2022 (sq. Km)	Area change (2002 - 2022)	Percent Change (%)
Sal_Mix forest	398.54	401.56	3.01	0.76
Teak_forest	70.78	28.84	-41.93	-59.25
Grassland	13.26	2.11	-11.15	-84.10
Degraded_forest	0.32	17.97	17.65	5546.62
Waterbodies	25.61	29.04	3.42	13.37
Cropland	316.46	280.53	-35.92	-11.35
Built-up	12.97	63.15	50.18	386.93
Floodplain_seasonal_stream	26.72	41.45	14.73	55.10

Table S 5: The LULC classes change between 2002 and 2022 in Block 1. (The top 10% of class changes are highlighted in bold font)

LULC Change_detection (2002-2022)	Area_change (sq. Km)
Sal_Mix_forest -Sal_Mix_forest	**410.91**
Sal_Mix_forest -Teak_forest	5.02
Sal_Mix_forest -Grassland	0.28
Sal_Mix_forest -Degraded_forest	**6.45**
Sal_Mix_forest -Waterbodies	1.45
Sal_Mix_forest -Cropland	4.96
Sal_Mix_forest -Built-up	1.34
Sal_Mix_forest -Floodplain_seasonal_stream	**9.82**
Teak_forest -Sal_Mix_forest	4.25
Teak_forest -Teak_forest	4.27
Teak_forest -Grassland	0.16
Teak_forest -Degraded_forest	0.20
Teak_forest -Waterbodies	0.16
Teak_forest -Cropland	0.13
Teak_forest -Built-up	0.13
Teak_forest -Floodplain_seasonal_stream	1.70
Grassland -Sal_Mix_forest	0.07
Grassland -Teak_forest	0.01
Grassland -Floodplain_seasonal_stream	0.01
Degraded_forest -Sal_Mix_forest	0.01
Degraded_forest -Floodplain_seasonal_stream	0.01

Waterbodies -Sal_Mix_forest	1.46
Waterbodies -Teak_forest	0.12
Waterbodies -Degraded_forest	0.16
Waterbodies -Waterbodies	2.46
Waterbodies -Cropland	0.07
Waterbodies -Built-up	0.22
Waterbodies -Floodplain_seasonal_stream	2.44
Cropland -Sal_Mix_forest	2.64
Cropland -Teak_forest	0.67
Cropland -Degraded_forest	0.67
Cropland -Waterbodies	0.26
Cropland -Cropland	**6.65**
Cropland -Built-up	0.41
Cropland -Floodplain_seasonal_stream	1.64
Built-up -Sal_Mix_forest	0.95
Built-up -Teak_forest	0.09
Built-up -Degraded_forest	0.02
Built-up -Waterbodies	0.23
Built-up -Cropland	0.20
Built-up -Built-up	0.33
Built-up -Floodplain_seasonal_stream	0.32
Floodplain_seasonal_stream -Sal_Mix_forest	**5.55**
Floodplain_seasonal_stream -Teak_forest	2.43
Floodplain_seasonal_stream -Grassland	0.08
Floodplain_seasonal_stream -Degraded_forest	0.89
Floodplain_seasonal_stream -Waterbodies	2.69
Floodplain_seasonal_stream -Cropland	1.83
Floodplain_seasonal_stream -Built-up	0.66
Floodplain_seasonal_stream -Floodplain_seasonal_stream	**11.67**

Table S 6: The LULC classes change between 2002 and 2022 in Block 2. (The top 10% of class changes are highlighted in bold font)

LULC Change_detection (2002-2022)	Area_change (sq. Km)
Sal_Mix forest -Sal_Mix forest	**300.16**
Sal_Mix forest -Teak forest	13.30
Sal_Mix forest -Grassland	1.18
Sal_Mix forest -Degraded forest	7.43
Sal_Mix forest -Waterbodies	5.21
Sal_Mix forest -Cropland	**46.50**
Sal_Mix forest -Built-up	14.78
Sal_Mix forest -Floodplain and seasonal streams	9.71
Teak forest -Sal_Mix forest	**43.71**
Teak forest -Teak forest	10.17
Teak forest -Grassland	0.53
Teak forest -Degraded forest	7.23
Teak forest -Waterbodies	0.30
Teak forest -Cropland	5.02
Teak forest -Built-up	1.72

**Teak forest -Floodplain and seasonal streams**	2.05
**Grassland -Sal_Mix forest**	7.11
**Grassland -Teak forest**	0.72
**Grassland -Grassland**	0.24
**Grassland -Degraded forest**	0.90
**Grassland -Waterbodies**	0.75
**Grassland -Cropland**	1.80
**Grassland -Built-up**	1.08
**Grassland -Floodplain and seasonal streams**	0.64
**Degraded forest -Sal_Mix forest**	0.11
**Degraded forest -Teak forest**	0.02
**Degraded forest -Degraded forest**	0.03
**Degraded forest -Waterbodies**	0.02
**Degraded forest -Cropland**	0.08
**Degraded forest -Built-up**	0.05
**Degraded forest -Floodplain and seasonal streams**	0.01
**Waterbodies -Sal_Mix forest**	5.40
**Waterbodies -Teak forest**	0.11
**Waterbodies -Degraded forest**	0.11
**Waterbodies -Waterbodies**	12.66
**Waterbodies -Cropland**	4.32
**Waterbodies -Built-up**	1.52
**Waterbodies -Floodplain and seasonal streams**	1.45
**Cropland -Sal_Mix forest**	**39.47**
**Cropland -Teak forest**	4.06
**Cropland -Grassland**	0.15
**Cropland -Degraded forest**	2.11
**Cropland -Waterbodies**	4.26
**Cropland -Cropland**	**213.95**
**Cropland -Built-up**	**37.40**
**Cropland -Floodplain and seasonal streams**	14.69
**Built-up -Sal_Mix forest**	2.62
**Built-up -Teak forest**	0.12
**Built-up -Degraded forest**	0.05
**Built-up -Waterbodies**	0.65
**Built-up -Cropland**	3.27
**Built-up -Built-up**	4.13
**Built-up -Floodplain and seasonal streams**	2.12
**Floodplain and seasonal streams -Sal_Mix forest**	2.75
**Floodplain and seasonal streams -Teak forest**	0.31
**Floodplain and seasonal streams -Degraded forest**	0.11
**Floodplain and seasonal streams -Waterbodies**	5.16
**Floodplain and seasonal streams -Cropland**	5.33
**Floodplain and seasonal streams -Built-up**	2.33
**Floodplain and seasonal streams -Floodplain and seasonal streams**	10.68

Table S 7: Goodness of Fit of GLM models for implications on different species hotspot (dependent variable) in both the study blocks. [Model variables: Habitat = (intercept), EVI_Change, NTL_Change, LULC_Change, and GLW_Cattle_density; Leopard = KDE_Hostpot of Leopard only; Tiger = KDE_Hostpot of tiger only; Large Carnivore = combined leopard and tiger KDE_Hostpot]

Study Blocks	Species (Dependent variable)	Models	Akaike's Information Criterion (AIC)	Finite Sample Corrected AIC (AICC)	Bayesian Information Criterion (BIC)
Block 1	Leopard	Habitat	175.365	175.788	190.351
		Habitat+Tiger	162.544	163.14	180.527
	Tiger	Habitat	1376.599	1376.809	1400.637
		Habitat+Leopard	1014.819	1015.181	1046.869
	Large Carnivore	Habitat	1619.672	1619.956	1647.665
		Habitat+Tiger+Leopard	1377.354	1377.812	1413.345
Block 2	Leopard	Habitat	88.032	88.321	115.884
		Habitat+Tiger	47.765	48.138	79.596
	Tiger	Habitat	-2196.471	-2196.27	-2159.804
		Habitat+Leopard	-2249.538	-2249.286	-2208.288
	Large Carnivore	Habitat	218.288	218.508	254.274
		Habitat+Tiger+Leopard	-128.684	-128.347	-83.702

Table S 8: Tests of model effects (selected GLM) of different factors and covariates in both the Study Blocks on conflict hotspot of leopard, tiger, and large carnivore (both tiger and leopard). (GLW_cattle_D denote cattle density) [EVI- Enhanced Vegetation Index, NTL- Night Time Light, GLW- Gridded Livestock of the World, LULC- Land Use Land Cover]

Study Blocks	Species (Dependent variable)	Models	Type-III	EVI_Change	NTL_Change	Tiger_KDE	Leopard_KDE	LULC_Change	GLW_Cattle_D
Block 1	Leopard	Habitat+Tiger	Wald Chi-Square	0.004	4.688	13.247	N/A	3.255	.a
			p-Value	0.948	**0.030**	**0.000**	N/A	0.071	.
	Tiger	Habitat+Leopard	Wald Chi-Square	6.669	1.026	N/A	31.393	5.691	1.466
			p-Value	**0.036**	0.311	N/A	**0.000**	**0.017**	0.226
	Large Carnivore	Habitat+Tiger+Leopard	Wald Chi-Square	0.495	0.568	106.989	10.232	0.032	8.027
			p-Value	0.781	0.451	**0.000**	**0.001**	0.857	**0.005**
Block 2	Leopard	Habitat+Tiger	Wald Chi-Square	1.878	18.128	41.654	N/A	1.438	0.307
			p-Value	0.171	**0.000**	**0.000**	N/A	0.230	0.579
	Tiger	Habitat+Leopard	Wald Chi-Square	0.174	7.406	N/A	46.806	3.138	30.807
			p-Value	0.917	**0.025**	N/A	**0.000**	0.077	**0.000**
	Large Carnivore	Habitat+Tiger+Leopard	Wald Chi-Square	5.457	0.328	2.820	265.168	0.976	14.625
			p-Value	0.065	0.849	0.093	**0.000**	0.323	**0.000**

a. Unable to compute due to numerical problems

Table S 9: Generalized Linear Model (GLM) parameter estimates of independent variables whose effects have been tested on negative Human-Leopard Interaction (HLI) hotspot in Study Block 1. (B is coefficient, sig. is p value for significance, df is degree of freedom, GLW_cattle denote cattle density) [EVI- Enhanced Vegetation Index, NTL- Night Time Light, GLW- Gridded Livestock of the World, LULC- Land Use Land Cover]

Parameter Estimates

Parameter	B	Std. Error	95% Wald Confidence Interval Lower	95% Wald Confidence Interval Upper	Hypothesis Test Wald Chi-Square	Hypothesis Test df	Hypothesis Test Sig.
(Intercept)	-1.070	.2152	-1.492	-.648	24.733	1	.000
[EVI_Change_block1=Greening]	.015	.2251	-.427	.456	.004	1	.948
[EVI_Change_block1=None]	0[a]	.	.	.	.	.	.
[NTL_Change_block1=Decrease]	-1.266	.5848	-2.412	-.120	4.688	1	**.030**
[NTL_Change_block1=No Change]	0[a]	.	.	.	.	.	.
Tiger_KDE_block1	.114	.0314	.053	.176	13.247	1	**.000**
LULC_Change_block1	.011	.0061	-.001	.023	3.255	1	.071
GLW_Cattle_D_block1	0[a]	.	.	.	.	.	.
(Scale)	1.619[b]	.1585	1.336	1.961			

Dependent Variable: Leopard_KDE_block1

Model: (Intercept), EVI_Change_block1, NTL_Change_block1, Tiger_KDE_block1, LULC_Change_block1, GLW_Cattle_D_block1

a. Set to zero because this parameter is redundant.

b. Maximum likelihood estimate.

Table S 10: Generalized Linear Model (GLM) parameter estimates of independent variables whose effects have been tested on negative Human-Tiger Interaction (HTI) hotspot in Study Block 1. (B is coefficient, sig. is p value for significance, df is degree of freedom, GLW_cattle denote cattle density) [EVI- Enhanced Vegetation Index, NTL- Night Time Light, GLW- Gridded Livestock of the World, LULC- Land Use Land Cover]

Parameter Estimates

Parameter	B	Std. Error	95% Wald Confidence Interval		Hypothesis Test		
			Lower	Upper	Wald Chi-Square	df	Sig.
(Intercept)	.300	.1436	.019	.582	4.377	1	.036
[EVI_Change_block1=Browning]	-.347	1.0999	-2.503	1.809	.100	1	.752
[EVI_Change_block1=Greening]	-.428	.1668	-.755	-.101	6.591	1	**.010**
[EVI_Change_block1=None]	0[a]	.	.	.	.	.	.
[NTL_Change_block1=Decrease]	.582	.5750	-.544	1.709	1.026	1	.311
[NTL_Change_block1=No Change]	0[a]	.	.	.	.	.	.
Leopard_KDE_block1	.871	.1554	.566	1.175	31.393	1	**.000**
LULC_Change_block1	.012	.0051	.002	.022	5.691	1	**.017**
GLW_Cattle_D_block1	.000	.0001	-8.184E-5	.000	1.466	1	.226
(Scale)	2.246[b]	.1288	2.008	2.513			

Dependent Variable: Tiger_KDE_block1

Model: (Intercept), EVI_Change_block1, NTL_Change_block1, Leopard_KDE_block1, LULC_Change_block1, GLW_Cattle_D_block1

a. Set to zero because this parameter is redundant.

b. Maximum likelihood estimate.

Table S 11: Generalized Linear Model (GLM) parameter estimates of independent variables whose effects have been tested on negative HLCI hotspot in Study Block 1. (B is coefficient, sig. is p value for significance, df is degree of freedom, GLW_cattle denote cattle density) [EVI- Enhanced Vegetation Index, NTL- Night Time Light, GLW- Gridded Livestock of the World, LULC- Land Use Land Cover]

Parameter Estimates

Parameter	B	Std. Error	95% Wald Confidence Interval Lower	95% Wald Confidence Interval Upper	Hypothesis Test Wald Chi-Square	Hypothesis Test df	Hypothesis Test Sig.
(Intercept)	-.141	.1369	-.409	.127	1.060	1	.303
[EVI_Change_block1=Browning]	.374	.9421	-1.4 72	2.221	.158	1	.691
[EVI_Change_block1=Greening]	-.083	.1428	-.362	.197	.334	1	.563
[EVI_Change_block1=None]	0[a]	.	.	.	.	.	.
[NTL_Change_block1=Decrease]	.328	.4357	-.526	1.182	.568	1	.451
[NTL_Change_block1=No Change]	0[a]	.	.	.	.	.	.
Tiger_KDE_block1	.337	.0326	.273	.401	106.989	1	**.000**
Leopard_KDE_block1	.400	.1249	.155	.645	10.232	1	**.001**
LULC_Change_block1	.001	.0044	-.008	.009	.032	1	.857
GLW_Cattle_D_block1	.000	.0001	9.729E-5	.001	8.027	1	**.005**
(Scale)	1.622[b]	.0962	1.444	1.822			

Dependent Variable: LC_KDE_block1

Model: (Intercept), EVI_Change_block1, NTL_Change_block1, Tiger_KDE_block1, Leopard_KDE_block1, LULC_Change_block1, GLW_Cattle_D_block1

a. Set to zero because this parameter is redundant.

b. Maximum likelihood estimate.

Table S 12: Generalized Linear Model (GLM) parameter estimates of independent variables whose effects have been tested on negative HLI hotspot in Study Block 2. (B is coefficient, sig. is p value for significance, df is degree of freedom, GLW_cattle denote cattle density) [EVI- Enhanced Vegetation Index, NTL- Night Time Light, GLW- Gridded Livestock of the World, LULC- Land Use Land Cover]

Parameter Estimates

Parameter	B	Std. Error	95% Wald Confidence Interval		Hypothesis Test		
			Lower	Upper	Wald Chi-Square	df	Sig.
(Intercept)	-1.295	.1396	-1.568	-1.021	86.035	1	.000
[EVI_Change_block2=Greening]	.341	.2487	-.147	.828	1.878	1	.171
[EVI_Change_block2=None]	0[a]	.	.	.	.	.	.
[NTL_Change_block2=Decrease]	-1.119	.2729	-1.654	-.585	16.830	1	**.000**
[NTL_Change_block2=Increase]	.301	.3787	-.441	1.043	.632	1	.427
[NTL_Change_block2=No Change]	0[a]	.	.	.	.	.	.
Tiger_KDE_block2	4.620	.7158	3.217	6.023	41.654	1	**.000**
LULC_Change_block2	-.004	.0032	-.010	.002	1.438	1	.230
GLW_Cattle_D_block2	2.174E-5	3.9224E-5	-5.514E-5	9.861E-5	.307	1	.579
(Scale)	1.597[b]	.0958	1.419	1.796			

Dependent Variable: Leopard_KDE_block2

Model: (Intercept), EVI_Change_block2, NTL_Change_block2, Tiger_KDE_block2, LULC_Change_block2, GLW_Cattle_D_block2

a. Set to zero because this parameter is redundant.

b. Maximum likelihood estimate.

Table S 13: Generalized Linear Model (GLM) parameter estimates of independent variables whose effects have been tested on negative HTI hotspot in Study Block 2. (B is coefficient, sig. is p value for significance, df is degree of freedom, GLW_cattle denote cattle density) [EVI- Enhanced Vegetation Index, NTL- Night Time Light, GLW- Gridded Livestock of the World, LULC- Land Use Land Cover]

Parameter Estimates

			95% Wald Confidence Interval		Hypothesis Test		
Parameter	B	Std. Error	Lower	Upper	Wald Chi-Square	df	Sig.
(Intercept)	-2.897	.0702	-3.035	-2.760	1702.249	1	.000
[EVI_Change_block2=Browning]	.428	1.0997	-1.728	2.583	.151	1	.697
[EVI_Change_block2=Greening]	.024	.1576	-.284	.333	.024	1	.877
[EVI_Change_block2=None]	0[a]	.	.	.	.	.	.
[NTL_Change_block2=Decrease]	.569	.2115	.155	.983	7.239	1	**.007**
[NTL_Change_block2=Increase]	-.084	.2807	-.634	.466	.090	1	.764
[NTL_Change_block2=No Change]	0[a]	.	.	.	.	.	.
Leopard_KDE_block2	.668	.0976	.476	.859	46.806	1	**.000**
LULC_Change_block2	-.004	.0022	-.008	.000	3.138	1	.077
GLW_Cattle_D_block2	.000	2.4162E-5	8.675E-5	.000	30.807	1	**.000**
(Scale)	1.206[b]	.0549	1.103	1.318			

Dependent Variable: Tiger_KDE_block2

Model: (Intercept), EVI_Change_block2, NTL_Change_block2, Leopard_KDE_block2, LULC_Change_block2, GLW_Cattle_D_block2

a. Set to zero because this parameter is redundant.

b. Maximum likelihood estimate.

Table S 14: Generalized Linear Model (GLM) parameter estimates of independent variables whose effects have been tested on negative HLCI hotspot in Study Block 2. (B is coefficient, sig. is *p* value for significance, df is degree of freedom, GLW_cattle denote cattle density) [EVI- Enhanced Vegetation Index, NTL- Night Time Light, GLW- Gridded Livestock of the World, LULC- Land Use Land Cover]

Parameter Estimates

Parameter	B	Std. Error	95% Wald Confidence Interval		Hypothesis Test		
			Lower	Upper	Wald Chi-Square	df	Sig.
(Intercept)	-1.833	.0972	-2.024	-1.643	355.591	1	.000
[EVI_Change_block2=Browning]	-.753	1.2422	-3.188	1.682	.367	1	.544
[EVI_Change_block2=Greening]	-.441	.1949	-.823	-.059	5.131	1	**.024**
[EVI_Change_block2=None]	0[a]	.	.	.	.	.	.
[NTL_Change_block2=Decrease]	-.026	.2540	-.524	.471	.011	1	.917
[NTL_Change_block2=Increase]	-.194	.3409	-.862	.474	.325	1	.569
[NTL_Change_block2=No Change]	0[a]	.	.	.	.	.	.
Tiger_KDE_block2	-1.064	.6338	-2.306	.178	2.820	1	.093
Leopard_KDE_block2	2.412	.1481	2.122	2.703	265.168	1	**.000**
LULC_Change_block2	.002	.0024	-.002	.007	.976	1	.323
GLW_Cattle_D_block2	.000	2.9760E-5	5.548E-5	.000	14.625	1	**.000**
(Scale)	1.537[b]	.0714	1.403	1.683			

Dependent Variable: LC_KDE_block2

Model: (Intercept), EVI_Change_block2, NTL_Change_block2, Tiger_KDE_block2, Leopard_KDE_block2, LULC_Change_block2, GLW_Cattle_D_block2

a. Set to zero because this parameter is redundant.

b. Maximum likelihood estimate.
